# Analysis and Validation of circRNA-miRNA Network in Regulating m^6^A RNA Methylation Modulators Reveals CircMAP2K4/miR-139-5p/YTHDF1 Axis Involving the Proliferation of Hepatocellular Carcinoma

**DOI:** 10.3389/fonc.2021.560506

**Published:** 2021-02-23

**Authors:** Fanwu Chi, Yong Cao, Yuhan Chen

**Affiliations:** ^1^ Cardiovascular Surgery Department, The People’s Hospital of Gaozhou, Gaozhou, China; ^2^ Department of Radiation Oncology, Nanfang Hospital, Southern Medical University, Guangzhou, China

**Keywords:** circular RNA, microRNA, m^6^A RNA methylation modulator, regulatory network, hepatocellular carcinoma

## Abstract

The m^6^A RNA methylation modulators play a crucial role in regulating hepatocellular carcinoma (HCC) progression. The circular RNA (circRNA) regulatory network in regulating m^6^A RNA methylation modulators in HCC remains largely unknown. In this study, 5 prognostic m^6^A RNA methylation modulators in HCC were identified from The Cancer Genome Atlas (TCGA) and International Cancer Genome Consortium (ICGC) projects. The differentially expressed microRNAs (DEmiRNAs) and circRNAs (DEcircRNAs) between paired tumor and normal tissues were screened out from TCGA and or Gene Expression Omnibus (GEO) database to construct the circRNA-miRNA- m^6^A RNA methylation modulator regulatory network, which included three m^6^A RNA methylation modulators (HNRNPC, YTHDF1, and YTHDF2), 11 DEmiRNAs, and eight DEcircRNAs. Among the network, hsa-miR-139-5p expression was negatively correlated with YTHDF1. Hsa-miR-139-5p low or YTHDF1 high expression was correlated with high pathological grade, advanced stage and poor survival of HCC. Additionally, cell cycle, base excision repair, and homologous recombination were enriched in YTHDF1 high expression group by GSEA. A hub circRNA regulatory network was constructed based on hsa-miR-139-5p/YTHDF1 axis. Furthermore, hsa_circ_0007456(circMAP2K4) was validated to promote HCC cell proliferation by binding with hsa-miR-139-5p to promote YTHDF1 expression. Taken together, we identified certain circRNA regulatory network related to m^6^A RNA methylation modulators and provided clues for mechanism study and therapeutic targets for HCC.

## Introduction

Hepatocellular carcinoma (HCC) is one of the most common malignant tumors worldwide, accounting for 75%–85% of primary liver cancer (1). Despite advances in diagnosis and therapeutic strategies in recent years, the prognosis of HCC is still not ideal. Therefore, there exists an urgent need to identify sensitive and specific biomarkers and therapeutic targets for the early diagnosis and treatment of HCC (2).

Among the chemical modification of RNA, N6-methyladenosine (m^6^A) methylated at the N6 position of adenosine is viewed as the most common, abundant and conservative internal transcriptional modification for various kinds of RNA. The m^6^A modifications are involved in RNA processing, transporting, translation and metabolism (3). Based on the different functions of m^6^A RNA methylation modulators, they are usually classified into “writers”, “erasers”, and “readers” (4). The “writers” catalyze the formation of m^6^A, including Methyltransferase-like 3 (METTL3) (5), METTL14 (6), Wilms tumor 1-associated protein (WTAP) (7), RNA Binding Motif Protein 15 (RBM15) (8) and KIAA1429 (9). The “erasers”, removing m^6^A modification from RNA, compose of fat mass and obesity-associated protein (FTO) (10) and alkB homologue 5 (ALKBH5) (11). The m^6^A readers YT521-B homology (YTH) domain-containing proteins (YTHDF1/2 and YTHDC1/2) function as m^6^A binding proteins that recognize m^6^A methylation and generate a functional signal (12). Accumulating evidence has demonstrated that m^6^A modifications participate in the progression of cancers, such as glioma, breast cancers and hepatocellular carcinoma (HCC) (13).

Circular RNA (circRNA) is a class of covalently closed single-stranded circular RNA molecules formed by back-splicing. CircRNA is considered as the RNA with tissue-, developmental stage- and disease-specificity (14). Notably, it is well documented that circRNAs play crucial roles in cancer by acting as microRNA (miRNA) sponge to modulate the miRNA-mRNA regulatory axis, thereby affecting the initiation and progression of cancer (15). However, whether circRNAs can serve as miRNA sponge to affect the miRNA in the regulation of m^6^A RNA methylation modulators in HCC has not been reported yet.

The flowchart of this study design was shown in **Figure S1**. Briefly, we identified prognostic m^6^A RNA methylation modulators in HCC patients from The Cancer Genome Atlas (TCGA) and International Cancer Genome Consortium (ICGC) projects. According to the differentially expressed (DE) circRNAs and miRNAs between paired normal and tumor tissues, the circRNA-miRNA-mRNA regulatory network was constructed based on the m^6^A RNA methylation modulators. Among the 3 co-expressed miRNA-m^6^A RNA methylation modulators pairs, hsa-miR-139-5p low or YTHDF1 high expression was significantly correlated with high pathological grade, advanced stage and poor survival of HCC. Therefore, a hub circRNA regulatory network was constructed based on hsa-miR-139-5p/YTHDF1 axis. Among this hub network, circMAP2K4 was validated to promote HCC cell proliferation by binding with hsa-miR-139-5p to promote YTHDF1 expression. These findings indicate certain circRNA regulatory network is involved in the regulation of m^6^A RNA methylation modulators and provide clues for mechanism study and therapeutic strategy development for HCC.

## Material and Methods

### Data Collection

Regarding the expression data of m6A RNA methylation modulators, we obtained transcriptome data of TCGA-LIHC project from TCGA data portal (https://tcga-data.nci.nih.gov/tcga/) and ICGC-LIRI-JP project from ICGC data portal (https://dcc.icgc.org/), respectively. Regarding the miRNA data, in order to maintain the consistency of data sources, we downloaded miRNA-seq data from the TCGA-LIHC project for subsequent difference and co-expression analysis. For the verification of the prognostic value of miRNA, we searched the Gene Expression Omnibus (GEO) database (http://www.ncbi.nlm.nih.gov/gds/) with “microRNA”, “hepatocellular carcinoma”, and “survival” as keywords. In order to ensure the reliability of the results, we only selected datasets with more than 100 cases for analysis, and finally included the GSE31384 into this study. Regarding the circRNA data, we searched the GEO database with “circular RNA”, “hepatocellular carcinoma”, and “microarray” as keywords, and finally included GSE94508, GSE97332 and GSE78520 into this study. Criteria for study inclusion were: 1) The disease was diagnosed as HCC. 2) HCC caused by different etiologies was acceptable. 3) The case had a complete expression profile. 4) The case had clinical information. Criteria for study exclusion were: 1) The survival data was unknown or survival time was less than 30 days. 2) The clinical staging and or pathological grade was unknown. The analysis flowchart of HCC cases with complete expression data was shown in **Figure S2**.

### Identification of DERNAs

The mRNA expression level of 13 m^6^A RNA methylation modulators were compared between 50 or 199 paired tumor and non-tumor samples from TCGA and ICGC projects by Mann-Whitney-Wilcoxon Test, respectively. And the prognostic value of the m^6^A RNA methylation modulators in both TCGA and ICGC were further assessed by univariate Cox regression survival analyses. Finally, those prognostic m^6^A RNA methylation modulators in both TCGA and ICGC were identified for the following network construction. The DEmiRNAs were screened out from 49 paired tumor and non-tumor samples from TCGA by using R package “Bioconductor Limma”. The adjusted P value (false discovery rate, FDR) of each gene was calculated by Benjamini Hochberg method and the threshold for DEmiRNA selection was FDR <0.05 and | log2FC |> 1. Finally, DEmiRNA is visualized by volcano graph and fold change (FC) filtering. According to the significance scores <0.01 and | log2FC |> 2, the DEcircRNAs between tumor and non-tumor cases from multiple studies was determined by a robust rank aggregation method (16). And the DEcircRNAs were visualized by heatmap.

### Construction of CircRNA-miRNA Network Involved in Regulating m^6^A RNA Methylation Modulators

The miRNAs potentially targeting m^6^A RNA methylation regulators were predicted by microRNA Data Integration Portal (miRDIP), which integrated more than 20 miRNA related databases for miRNA target or miRNA prediction (17). Among the DEmiRNAs, the potential miRNAs targeting m^6^A RNA methylation regulators were selected with the very high score (top 1%) in miRDIP. Next, the DEcircRNAs targeted miRNAs were predicted by Cancer-Specific CircRNA Database (CSCD, https://http://gb.whu.edu.cn/CSCD/). As the sponge of miRNA, the expression level of circRNA usually does not influence the expression of miRNA. In addition, some miRNAs may inhibit highly expressed mRNA in a compensatory elevated expression manner (18). Therefore, the selection of circRNA-miRNA or miRNA-mRNA pairs was not limited by their expression patterns that must be reversed. Finally, the circRNA-miRNA-mRNA regulatory network was constructed after taking the intersection of DEcircRNA-miRNA pairs and DEmiRNA–m^6^A RNA methylation regulator pairs. The regulatory network was visualized using Cytoscape 3.4.0 (http://cytoscape.org/).

### Gene Set Enrichment Analysis (GSEA)

The HCC samples from TCGA or ICGC were divided into high- and low-expression groups according to the expression level of YTHDF1, respectively. GSEA (http://software.broadinstitute.org/gsea/index.jsp) was carried out to compare the potential biological pathways between two groups. The annotated gene set list c2.cp.kegg. v5.2.symbols.gmt was utilized as the reference gene set. The cut-off criteria were defined as FDR < 0.25 and a nominal *P* < 0.01. The gene sets with top 5 normalized enrichment score (NES) in high- and low-expression groups were selected for visualization.

### Cell Culture and Transfection

Human HCC cell lines Huh7, Hep3B, MHCC97H, HCCLM3, and normal LO2 cells were gained from Shanghai Advanced Research Institute, Chinese Academy of Sciences. Cells were cultured in Dulbecco’s Modified Eagle’s Medium (DMEM) (pH 7.4) supplemented with 10% (v/v) fetal bovine serum (Gibco). YTHDF1 siRNA, hsa-miR-139-5p mimics, circRNA overexpressing plasmid and their corresponding negative control were purchased from GenePharma (Shanghai, China). The cells in 24-well plates were transfected with 1ug plasmid, 50 nM mimics or siRNA by using Lipofectamine 3000 reagent (Invitrogen) according to the manufacturer’s recommendation. The specific siRNA sequences for YTHDF1 were provided in the **Table S1**. Three independent experiments were carried out for cell transfection.

### Quantitative Reverse Transcription Polymerase Reaction (qRT-PCR) Analysis

Total RNA were isolated from cells using TRIzol reagent (Invitrogen) and cDNA were synthesized by utilizing the Prime Script RT reagent kit (Takara Bio, Shiga, Japan). The SYBR^®^ Premix Ex Taq™ (Takara) were used for qRT-PCR detection through real-time detection system (ABI7500, USA). The primer sequences for detection were provided in **Table S2**. GAPDH was used as an internal standard control. Gene expression level was quantified using 2^-△△Ct^ method. The results were obtained from three independent experiments.

### Western Blot

Protein was extracted using RIPA (Beyotime, China) and separated on 10% SDS-PAGE gels and transferred onto polyvinylidene fluoride membranes (Millipore, USA). The primary antibodies of anti-YTHDF1 (#86463) and anti-GAPDH (#2118) were purchased from Cell Signaling Technology (Danvers, MA, USA). After incubating with the primary antibodies at 4°C overnight, the membranes were then subjected to HRP-conjugated secondary antibody (Cell Signaling Technology, USA) at room temperature for 1 h. The blots were visualized using an imaging system (Bio-Rad, USA).

### Agarose Gel Electrophoresis Analysis

Total RNA isolation and cDNA synthesis were mentioned as above. Total DNA was extracted using the SteadyPure Universal Genomic DNA Extraction Kit (Accurate Biology Co. Ltd., Changsha, China). The specific divergent primers and convergent primers for circMAP2K4 were used for amplification. The primer sequences were listed in **Table S2**. Then the amplification products were detected in 2% agarose gel electrophoresis. And those products amplified by divergent primers were used for Sanger sequencing to detect the junction site of circMAP2K4. Three independent experiments were carried out for agarose gel electrophoresis analysis.

### Cell Proliferation Assay

The transfected cells were seeded in 96-well plates at a density of 2,000 cells per well. Cell viability was accessed from 12 to 120 h by using the Cell Counting Kit-8 (CCK-8) according to the manufacturer’s recommendation (Dojindo, Kumamoto, Japan). The optical density (OD) was recorded at 450 nm by an automatic microplate reader (Synergy4; BioTek, Winooski, VT, USA). The results were obtained from three independent experiments.

### Luciferase Reporter Assay

The wild type or mutated circRNA sequence containing hsa-miR-139-5p binding site, the wild type or the mutated 3’ untranslated region (UTR) of YTHDF1, were respectively synthesized and inserted into pmiR-RB-REPORT™ vector (RIBOBIO, Guangzhou, China). The above vectors and hsa-miR-139-5p mimics or negative control were co-transfected into cells using Lipofectamine3000 (Invitrogen). 48 h after transfection, the cells were harvested for firefly and renilla luciferase activities detection by using the dual-luciferase reporter assay system (Promega, Massachusetts, USA). Renilla luciferase served as the internal control for luciferase activity. The results were obtained from three independent experiments.

### Statistical Analysis

OS differences between high and low expression groups were evaluated by Kaplan-Meier survival analysis and log-rank test. The differences of gene expression between each clinicopathological characteristics were evaluated by Mann-Whitney-Wilcoxon Test. Data differences between in-vitro experimental groups were analyzed by Student’s t-test or one-way analysis of variance (ANOVA). All tests were analyzed using R software version 3.4.2 and *P* < 0.05 was considered statistically significant.

## Results

### Most m^6^A RNA Methylation Modulators Are Up-Regulated and Correlated With the Prognosis of HCC

**Figure 1:**
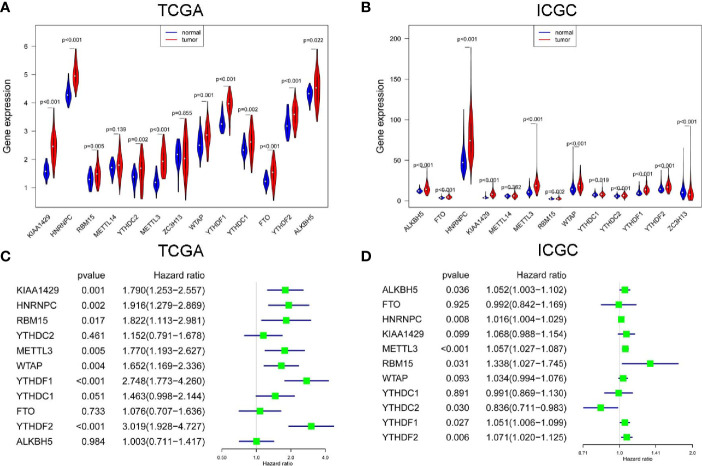
The expression and prognostic value of m6A RNA methylation modulators in HCC. The expression of m6A RNA methylation modulators in paired hepatocellular carcinoma (HCC) and normal tissues from The Cancer Genome Atlas (TCGA) **(A)** and International Cancer Genome Consortium (ICGC) **(B)**. The univariate Cox regression analysis of m6A RNA methylation modulators for OS of HCC cases from TCGA **(C)** and ICGC **(D)**.

In TCGA project, all m^6^A RNA methylation modulators were up-regulated in HCC, but the expression difference of METL14 and ZC3H13 were not statistically significant (**Figure 1A**). In ICGC project, most m^6^A RNA methylation modulators except ZC3H13 were up-regulated in HCC, but the up-regulation of METTL14 was not statistically different (**Figure 1B**). Next, we analyzed the prognostic values of 11 commonly DE m^6^A RNA methylation modulators in both TCGA and ICGC. High expression of YTHDF2, YTHDF1, KIAA1429, HNRNPC, WTAP, METTL3, or RBM15 was correlated with the poor survival of HCC in TCGA project by univariate Cox regression survival analysis (**Figure 1C**). While in ICGC project, high expression of METTL3, YTHDF2, HNRNPC, YTHDF1, YTHDC2, RBM15, or ALKBH5 was associated with poor survival of HCC (**Figure 1D**). Thus, the commonly prognostic m^6^A RNA methylation modulators, namely METTL3, YTHDF2, HNRNPC, YTHDF1 and RBM15, in both TCGA and ICGC were used for the following study.

### Construction of CircRNA-miRNA- m^6^A RNA Methylation Modulator Regulatory Network in HCC

**Figure 2:**
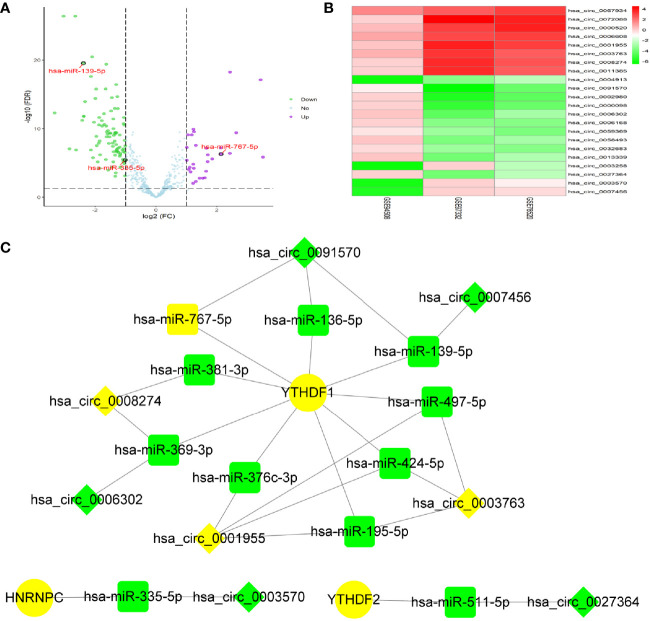
Identification of DEmiRNAs and DEcircRNAs for network construction. **(A)** The DEmiRNAs were screened out from The Cancer Genome Atlas (TCGA). **(B)** The DEcircRNAs were screened out from three GEO datasets. **(C)** Construction of circRNA-miRNA-mRNA network based on m6A RNA methylation modulators with prognostic value. The diamond, rectangle and ellipse indicated circRNA, miRNA, and mRNA, respectively. Yellow and light green represented up- and down-regulated, respectively.

By screening of miRNA-seq data from paired tumor and adjacent non-tumor tissues in TCGA HCC cases, a total of 121 DEmiRNAs (29 up and 92 down) were obtained (**Figure 2A**). From the circRNA microarray data of paired tumor and adjacent non-tumor tissues in 3 GEO datasets, a total of 22 DEcircRNAs (eight up and 14 down) were identified (**Figure 2B**). 209 miRNA-m^6^A RNA methylation modulators pairs and 1260 circRNA-miRNA pairs were predicted by miRDIP and CSCD, respectively. After taking the intersection of these RNA pairs, 3 m^6^A RNA methylation modulators (HNRNPC, YTHDF1, and YTHDF2), 11 DEmiRNAs, and eight DEcircRNAs were utilized to construct a circRNA-miRNA-m^6^A RNA methylation modulator regulatory network. This network contained 16 circRNA-miRNA pairs and 11 miRNA-mRNA pairs (**Figure 2C**). In order to verify the expression stability of DEmiRNAs and DEmRNAs in the regulatory network, we further analyzed the expression of DEmiRNAs in HCC by using dbDEMC 2.0, a database of differentially expressed miRNAs in human cancers (19) and the expression of DEmRNAs in HCC by using HCCDB, a database of hepatocellular carcinoma expression atlas (20), respectively. The results showed that most DEmiRNAs had the same expression trend in multiple GEO datasets, which were consistent with the results from TCGA and ICGC (**Table S3**). Especially, hsa-miR-139-5p was down-regulated in HCC tissues from even six datasets. Similarly, in accordance with the TCGA and ICGC results, the expression of YTHDF1, HNRNPC or YTHDF2 was up-regulated in HCC from seven, six, or four datasets, respectively (**Table S4**).

### Co-Expression and Clinicopathological Characteristics Correlation Analysis of miRNA

**Figure 3:**
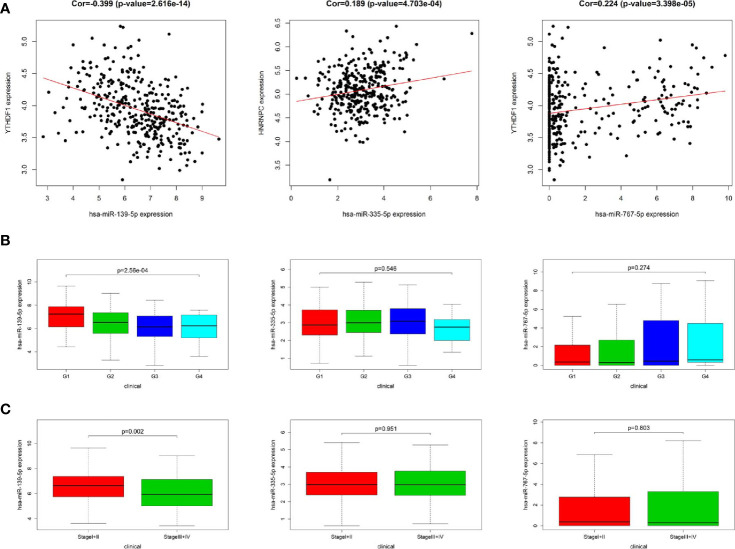
Relationship between miRNAs and m6A RNA methylation modulators as well as clinicopathological characteristics in The Cancer Genome Atlas (TCGA). **(A)** Co-expression analysis between three miRNAs and two m6A RNA methylation modulators. **(B)** Comparison of three miRNAs expression level between different pathological grades. **(C)** Comparison of three miRNAs expression level between different stages.

In order to identify the most potentially interactive miRNA-mRNA pairs, co-expression status between 11 DEmiRNAs and 3 m^6^A RNA methylation modulators were performed by Pearson correlation analysis. Three co-expressed miRNA-m^6^A RNA methylation modulator pairs were identified. As shown in **Figure 3A**, hsa-miR-139-5p expression was negatively correlated with YTHDF1 (r=-0.399, *P*<0.001), while hsa-miR-335-5p was positively correlated with HNRNPC (r=0.189, *P*<0.001) and hsa-miR-767-5p was positively correlated with YTHDF1 (r=0.224, *P*<0.001). Additionally, high expression of hsa-miR-139-5p but neither hsa-miR-335-5p nor hsa-miR-767-5p was significantly correlated with the low pathological grade in TCGA project (**Figure 3B**). Similarly, the higher the hsa-miR-139-5p expression, the earlier the TNM stage (**Figure 3C**).

### Clinicopathological Characteristics Correlation and GSEA of m^6^A RNA Methylation Modulators

**Figure 4:**
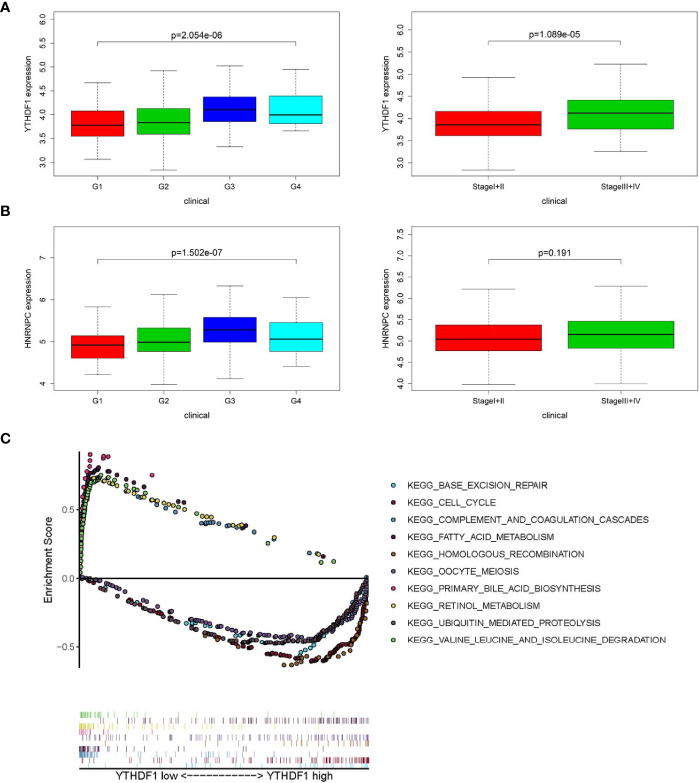
Relationship between m6A RNA methylation modulators and clinicopathological characteristics and gene set enrichment analysis (GSEA) in The Cancer Genome Atlas (TCGA). **(A)** Comparison of YTHDF1 expression level between different pathological grades and stages. **(B)** Comparison of HNRNPC expression level between different pathological grades and stages. **(C)** GSEA results showing the top five gene sets based on normalized enrichment score in YTHDF1 high or low expression groups.

In accordance with the negative correlation of hsa-miR-139-5p and YTHDF1 expression, the expression of YTHDF1 were higher in high pathological grade and advanced TNM stage in TCGA project (**Figure 4A**). While the expression of HNRNPC were significant different between different pathological grades but not between early and advanced TNM stage in TCGA project (**Figure 4B**). The relationships between YTHDF1 or HNRNPC expression and clinicopathological characteristics could not be fully investigated due to the lack of data about pathological grade in ICGC project. The expression of YTHDF1 or HNRNPC was significantly higher in the advanced TNM stage of HCC from ICGC project (**Figure S3A**). Based on the clinical significance of YTHDF1 in both TCGA and ICGC, we further performed GSEA to explore whether biological pathways differ between high and low YTHDF1 expression groups. Among top five gene sets based on NES, cell cycle, base excision repair and homologous recombination were enriched in YTHDF1 high expression group in both TCGA and ICGC (**Figure 4C**, **Figure S3B**). While fatty acid metabolism, retinol metabolism, complement and coagulation cascades were enriched in YTHDF1 low expression group in both TCGA and ICGC (**Figure 4C**, **Figure S3B**).

### Prognostic Value of the hsa-miR-139-5p and YTHDF1 Signature and Hub circRNA Network Construction

**Figure 5:**
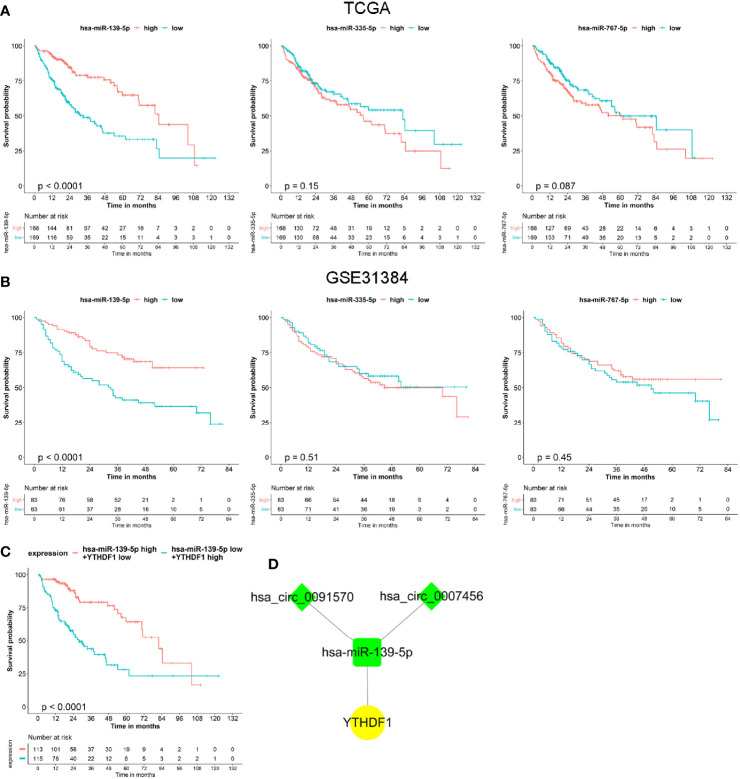
The prognostic value of miRNAs and hub circRNA regulatory network construction in hepatocellular carcinoma (HCC). **(A)** The Kaplan–Meier survival analysis of HCC cases with different miRNA expression level in The Cancer Genome Atlas (TCGA). **(B)** The Kaplan–Meier survival analysis of HCC cases with different miRNA expression level in GSE31384. **(C)** The Kaplan–Meier survival analysis of HCC cases with different expression level of hsa-miR-139-5p and YTHDF1 in TCGA. **(D)** Construction of hub circRNA regulatory network based on hsa-miR-139-5p/YTHDF1 axis. The diamond, rectangle and ellipse indicated circRNA, miRNA, and mRNA, respectively. Yellow and light green represented up- and down-regulated, respectively.

Based on the co-expressed results of miRNA and m^6^A RNA methylation modulators, we further explored the prognostic value of 3 miRNA. Survival analysis revealed that only the hsa-miR-139-5p expression status was correlated with the OS for HCC patients (**Figure 5A**). Similar results were observed in GSE31384 (**Figure 5B**). According to the results that hsa-miR-139-5p high or YTHDF1 low expression was associated with the better OS of HCC, we further evaluated the prognosis of HCC patients with hsa-miR-139-5p high and YTHDF1 low expression. The results showed that HCC patients with hsa-miR-139-5p high and YTHDF1 low expression had longer OS time than those with contrast expression level (**Figure 5C**). Based on the clinical significance of hsa-miR-139-5p and YTHDF1, a hub circRNA-miRNA-mRNA regulatory network was constructed finally. This hub network contained two regulatory axes, namely hsa_circ_0007456/hsa-miR-139-5p/YTHDF1 and hsa_circ_0091570/hsa-miR-139-5p/YTHDF1 (**Figure 5D**).

### CircMAP2K4 Promotes HCC Proliferation by Modulating hsa-miR-139-5p/YTHDF1

**Figure 6:**
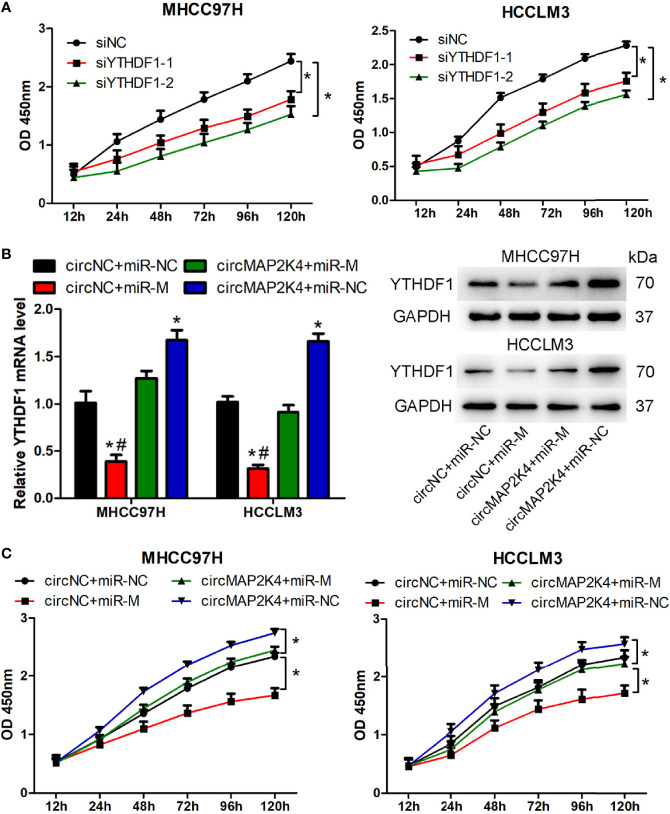
Overexpression of hsa_circ_007456(circMAP2K4) reverses the inhibitory effects of miR-139-5p on the proliferation of hepatocellular carcinoma (HCC) cells. **(A)** CCK8 assay showed the proliferation of HCC cells transfected with YTHDF1 siRNAs or negative control. *P < 0.05 vs. negative control. **(B)** qRT-PCR and western blot analysis of YTHDF1 in HCC cells at 24 h after transfection with circMAP2K4 expressing plasmid and or miR-139-5p mimics. *P < 0.05 vs. negative control, ^#^ *P <*0.05 vs. circMAP2K4+miR-M group. **(C)** CCK8 assay showed the proliferation of HCC cells transfected with circMAP2K4 expressing plasmid and or miR-139-5p mimics. siNC, siRNA negative control for YTHDF1; siYTHDF1: siRNA specifically against YTHDF1, miR-NC: miRNA negative control, miR-M: miRNA mimics, circNC: negative control for circMAP2K4 expressing plasmid. *P < 0.05 vs. negative control.

Two potentially dysregulated circRNAs were predicted to involve in regulating hsa-miR-139-5p/YTHDF1 axis in this study. We further analyzed the potential interaction of circRNA and miRNA through miRanda v3.3a, a microRNA target scanning algorithm. The result showed that hsa_circ_0007456 is predicted to has a score of 140 and energy of -19.98 kCal/Mol to interact with hsa-miR-139-5p, which is favorable for hsa_circ_0007456 serving as hsa-miR-139-5p sponge. But no predicting results were provided for the interaction of hsa_circ_0091570 and hsa-miR-139-5p. Thus, we selected the hsa_circ_0007456 for the following study. Hsa_circ_0007456 (circMAP2K4) derived from mitogen activated protein kinase kinase 4 (MAP2K4) gene and its position is located in chr17:11984672-12016677. The expression of circMAP2K4 or hsa-miR-139-5p was the highest in Huh7; moderate in Hep3B and MHCC97H; and lowest in HCCLM3 (**Figure S4A**). In contrast, the mRNA and protein expression of YTHDF1 were the highest in HCCLM3; moderate in Hep3B and MHCC97H; and lowest in Huh7 (**Figure S4A**). Agarose gel electrophoresis results showed that the amplified product of divergent primers for circMAP2K4 could be detected in cDNA but not in gDNA. (**Figure S4B**). Sanger sequencing also confirmed the junction site of circMAP2K4 provided by Circular RNA Interactome (**Figure S4C**). These results indicated that circMAP2K4 was a covalently closed-loop RNA. In order to explore the function of YTHDF1, two siRNAs were designed to knockdown the expression of YTHDF1 in MHCC97H and HCCLM3 cells. The results showed that these two siRNAs could effectively decrease the mRNA and protein expression of YTHDF1 in MHCC97H and HCCLM3 cells (**Figure S4D**). Transfection of YTHDF1 siRNAs significantly inhibited the proliferation of MHCC97H and HCCLM3 cells (**Figure 6A**). Next, we explored the function of circMAP2K4 on regulating hsa-miR-139-5p/YTHDF1 axis. Transfection of hsa-miR-139-5p mimics or circMAP2K4 expressing plasmid could effectively increase the expression of hsa-miR-139-5p (**Figure S4E**) or circMAP2K4 (**Figure S4F**), respectively. The luciferase assay showed that the luciferase activity was inhibited when co-transfection of hsa-miR-139-5p mimics and reporter plasmids containing circMAP2K4 wide type sequence with hsa-miR-139-5p binding site. While the luciferase activity had no obvious change when co-transfection of hsa-miR-139-5p mimics and reporter plasmids containing circMAP2K4 mutant sequence (**Figure S4G**). Using the miRanda algorithm, we found that YTHDF1 contains miRNA response element of hsa-miR-139-5p. Similarly, the luciferase activity of YTHDF1 wide type reporter plasmids was significantly inhibited by transfection of hsa-miR-139-5p mimics (**Figure S4G**). Transfection of hsa-miR-139-5p mimics significantly down-regulated the mRNA and protein expression of YTHDF1 in HCC cells, whereas these effects were reversed by circMAP2K4 overexpression (**Figure 6B**). Moreover, enforced hsa-miR-139-5p expression significantly inhibited the proliferation of HCC cells. However, additive circMAP2K4 overexpression partly abrogated the inhibitory effect of hsa-miR-139-5p on cell proliferation (**Figure 6C**). These results suggested that circMAPK4 acts as has-miR-139-5p sponge to regulate the expression and activity of YTHDF1.

## Discussion

The m^6^A RNA modification is a dynamic and reversible process, which is related to various diseases such as obesity, infertility and cancer (21). Numerous studies have confirmed that circRNAs act as miRNA sponges to modulate the pathogenesis of cancer, which facilitates them to serve as diagnostic and prognostic biomarkers and even therapeutic targets for tumors, including HCC (22). In the present study, we demonstrated that most m^6^A RNA methylation modulators are up-regulated and correlated with the prognosis of HCC. Based on the prognostic m^6^A RNA methylation modulators, a circRNA-miRNA-mRNA regulatory network was constructed. Among this network, hsa-miR-139-5p/YTHDF1 axis was illustrated to be associated with clinicopathological characteristics and prognosis of HCC. Moreover, circMAP2K4 could serve as the hsa-miR-139-5p sponge to up-regulate YTHDF1 expression and promote HCC proliferation.

In this study, METTL3, YTHDF2, HNRNPC, YTHDF1, and RBM15 were identified as the commonly prognostic m^6^A RNA methylation modulators for HCC in both TCGA and ICGC projects. Finally, 3 m^6^A RNA methylation modulators, namely HNRNPC, YTHDF1, and YTHDF2, were included for the construction of circRNA regulatory network. Our findings were consistent with previous reports that high expression of YTHDF1, HNRNPC, or METTL3 is related to the poor prognosis of HCC (23–25). While high level of METTL3 or YTHDF2 can be used as the poor prognostic factor for hepatoblastoma (26). In addition, the combination of YTHDF1 and METTL3 can reflect the malignant degree and evaluate the prognosis of HCC (27). Combining the biomarkers reported in the previous reports and the prognostic m^6^A RNA methylation modulators found in this study, we may try to construct a predictive signature related to the prognosis of HCC in the future study. This may be more beneficial to the evaluation of the prognosis of patients with HCC.

Accumulating evidence revealed that m^6^A writers, erasers, and readers participate in the development and progression of HCC by targeting various tumor-related genes. For example, overexpression of METTL3 promotes cell proliferation, migration, and clonal formation by inhibiting suppressor of cytokine signaling 2 mRNA expression in a m^6^A-YTHDF2-dependent manner (28). RAD52 motif 1 (RDM1) binds to the tumor suppressor p53 and enhances its stability, while METTL3 overexpression can significantly reduce the expression of RDM1 mRNA through m^6^A modification (24). In addition, knockdown of METTL3 results in the down-regulation of Snail, a key transcription factor of epithelial-mesenchymal transition (EMT), thereby reducing the invasion and EMT of HCC cell lines (29). As an m^6^A reader, YTHDF is also involved in the occurrence of HCC. YTHDF1 promotes HCC progression by enhancing FZD5 mRNA translation or AKT/GSK-3β/β-catenin signaling activation (30, 31). YTHDF2 is involved in the decay of IL11 and Serpine2 mRNA, which are important genes that regulate the normalization and inflammation of vessels (32). The expression of YTHDF2 in HCC is specifically induced by hypoxia, and overexpression of YTHDF2 inhibits cell proliferation, tumor growth and the activation of MEK and ERK. Mechanistically, YTHDF2 can bind to the 3′UTR m^6^A modification site of EGFR, and down-regulate the expression of EGFR mRNA in HCC cells (33). However, the specific mechanisms of HNRNPC in the development of HCC remain unclear. In this study, GSEA results showed that cell cycle, base excision repair and homologous recombination were enriched in YTHDF1 high expression group. Emerging studies had showed that cell cycle dysregulation and DNA damage repair are involved in HCC progression (34, 35), which indicates that high expression of YTHDF1 may promote the progression of HCC by regulating cell cycle progression and DNA damage repair. We also confirmed that knockdown the expression of YTHDF1 could inhibit the proliferation of HCC. These findings explain to some extent the reasons for high YTHDF1 expression was associated with high pathological grade, advanced TNM stage and poor survival of HCC in both TCGA and ICGC project. Combining the reported results with our findings indicate that m^6^A RNA methylation modulators play important roles in the occurrence and development of HCC.

Previous studies have demonstrated that some miRNAs participate in the regulation of RNA methylation modulators. For instance, hsa-miR-145 and YTHDF2 mRNA levels were negatively correlated in HCC tissues and overexpression of hsa-miR-145 could down-regulate the expression of YTHDF2, thereby increasing the mA level in HCC cells (36). In hepatoblastoma, METTL3 is identified as a direct target of hsa-miR-186, and the hsa-miR-186/METTL3 axis participates in the progress of hepatoblastoma through the Wnt/β-catenin signaling pathway (26). In this study, YTHDF1 was predicted to be the target gene of hsa-miR-139-5p and hsa-miR-767-5p with a co-expression relationship, and HNRNPC was co-expressed with and predicted to be targeted by hsa-miR-335-5p. There are no reports about these miRNAs acting on these m^6^A regulators right now. However, all three miRNAs have been shown to be involved in the development of HCC. For example, high expression of hsa-miR-139-5p and hsa-miR-335-5p can inhibit the proliferation and invasion of HCC cells and induce tumor shrinkage (37, 38). While HCC cells with hsa-miR-767-5p overexpression have significantly higher proliferation, migration and invasion potential (39). In addition, many studies have confirmed that high expression of hsa-miR-139-5p is associated with a better prognosis of HCC (37, 40). Different from previous studies, we firstly found that hsa-miR-139-5p could act on m^6^A RNA methylation modulator to regulate the progress of HCC. The present study demonstrated that the expression of hsa-miR-139-5p is negatively correlated with YTHDF1. High expression of hsa-miR-139-5p is associated with a lower grade, an earlier clinical stage, and a better prognosis of HCC, while high expression of YTHDF1 shows the contrast relationship. Moreover, in-vitro experiments also demonstrated that overexpression of hsa-miR-139-5p could inhibit the proliferation of HCC by targeting YTHDF1. These findings suggest that hsa-miR-139-5p/YTHDF1 regulatory axis play an important role in the development of HCC.

According to the important role of hsa-miR-139-5p/YTHDF1 regulatory axis in the progression of HCC, we further evaluated the candidate circRNAs involving in regulating hsa-miR-139-5p and hsa_circ_0007456 and hsa_circ_0091570 were identified finally. As for hsa_circ_0091570, it serves as hsa-miR-1307 sponge and its inhibition promotes HCC cell proliferation, migration and tumor growth in the mouse xenograft model (41). However, to date, the function of hsa_circ_0007456 (circMAP2K4) has not been reported in HCC, which encourages us to test whether circMAP2K4 can also function as miRNA sponge like hsa_circ_0091570 in regulating the proliferation of HCC. We firstly demonstrated that circMAP2K4, hsa-miR-139-5p and YTHDF1 participate in regulating the proliferation of HCC. Importantly, we validated that circMAP2K4 has the bind site for hsa-miR-139-5p and could reverse the repression of hsa-miR-139-5p on YTHDF1, thus eliminating the inhibitory effect of hsa-miR-139-5p on cell proliferation. YTHDF1 high expression was correlated with high pathological grade and advanced stage, which indicates YTHDF1 may be involved in the migration and metastasis of HCC. Indeed, previous studies have confirmed that upregulation of YTHDF1 improve the migratory and invasive capabilities of HCC cells (30, 31), which provide clues for the investigation of circMAP2K4/miR-139-5p/YTHDF1 axis in the migration and metastasis of HCC in our future study.

There are several limitations in this study. First, the datasets included in this study were from different sources, in which the circRNA data were obtained from the microarray data of GEO, while the data of miRNA and m^6^A RNA methylation modulators were obtained from the sequencing data of TCGA or ICGC. Different data sources may affect the reliability of the conclusions to a certain extent. There was no survival information related to the circRNA microarray of HCC in GEO database and due to our lack of HCC tissues, we were unable to evaluate the prognostic value of circRNAs for HCC. Second, due to the limited datasets included in this study, it may lead to that these identified DERNAs were not the most representative although we have verified the expression status of DERNAs through different databases. Moreover, with the advancement of technology and research, more and more m^6^A RNA methylation modulators are discovered. In this study, only 13 m^6^A RNA methylation modulators were included for analysis, and the threshold for circRNA selection was relatively strict, which may lead to the loss of some DEcircRNAs and m^6^A RNA methylation modulators.

In conclusion, we utilize the m^6^A RNA methylation modulators with prognostic value combined with DEcircRNAs and DEmiRNAs to construct a circRNA regulatory network in HCC. Among this network, the expression of hsa-miR-139-5p was negatively correlated with YTHDF1. hsa-miR-139-5p low or YTHDF1 high expression was illustrated to be associated with high grade, advanced stage and poor prognosis of HCC. The hub circRNA regulatory network was constructed based on hsa-miR-139-5p/YTHDF1 axis. In the hub network, circMAP2K4 could serve as the hsa-miR-139-5p sponge to up-regulate YTHDF1 expression and promote HCC proliferation. Our findings indicate that certain circRNA regulatory network is involved in the regulation of m^6^A RNA methylation modulators and provide a novel insight into mechanism study and therapeutic targets for HCC.

## Data Availability Statement

Publicly available data sets were analyzed in this study. These data can be found here: TCGA; ICGC; GSE31384; GSE94508; GSE97332; GSE78520.

## Author Contributions

YuC designed the study and conducted the experiment. FC performed the specific procedures and wrote the manuscript. YoC analyzed the data and made the pictures and graphs. All the authors have read and approved the manuscript. All authors contributed to the article and approved the submitted version.

## Funding

This study was supported by Natural Science Foundation of Guangdong Province (grant no. 2019A1515011652), Outstanding Youth Development Scheme of Nanfang Hospital, Southern Medical University (grant no. 2019J006), President Foundation of Nanfang Hospital, Southern Medical University (grant no. 2018C001), and National Natural Science Foundation of China (grant no. 81903132).

## Conflict of Interest

The authors declare that the research was conducted in the absence of any commercial or financial relationships that could be construed as a potential conflict of interest.

## References

[1] Bray F Ferlay J Soerjomataram I Siegel RL Torre LA Jemal A. Global cancer statistics 2018: GLOBOCAN estimates of incidence and mortality worldwide for 36 cancers in 185 countries. CA: Cancer J Clin (2018) 68(6):394–424. 10.3322/caac.21492 30207593

[2] Yang JD Hainaut P Gores GJ Amadou A Plymoth A Roberts LR. A global view of hepatocellular carcinoma: trends, risk, prevention and management. Nat Rev Gastroenterol Hepatol (2019) 16(10):589–604. 10.1038/s41575-019-0186-y 31439937 PMC6813818

[3] Roundtree IA Evans ME Pan T He C. Dynamic RNA Modifications in Gene Expression Regulation. Cell (2017) 169(7):1187–200. 10.1016/j.cell.2017.05.045 PMC5657247 28622506

[4] He L Li H Wu A Peng Y Shu G Yin G. Functions of N6-methyladenosine and its role in cancer. Mol Cancer (2019) 18(1):176. 10.1186/s12943-019-1109-9 31801551 PMC6892141

[5] Zeng C Huang W Li Y Weng H. Roles of METTL3 in cancer: mechanisms and therapeutic targeting. J Hematol Oncol (2020) 13(1):117. 10.1186/s13045-020-00951-w 32854717 PMC7457244

[6] Weng H Huang H Wu H Qin X Zhao BS Dong L. METTL14 Inhibits Hematopoietic Stem/Progenitor Differentiation and Promotes Leukemogenesis via mRNA m(6)A Modification. Cell Stem Cell (2018) 22(2):191–205.e9. 10.1016/j.stem.2017.11.016 29290617 PMC5860916

[7] Ping XL Sun BF Wang L Xiao W Yang X Wang WJ. Mammalian WTAP is a regulatory subunit of the RNA N6-methyladenosine methyltransferase. Cell Res (2014) 24(2):177–89. 10.1038/cr.2014.3 PMC3915904 24407421

[8] Xie Y Castro-Hernández R Sokpor G Pham L Narayanan R Rosenbusch J. RBM15 Modulates the Function of Chromatin Remodeling Factor BAF155 Through RNA Methylation in Developing Cortex. Mol Neurobiol (2019) 56(11):7305–20. 10.1007/s12035-019-1595-1 31020615

[9] Hu Y Ouyang Z Sui X Qi M Li M He Y. Oocyte competence is maintained by m(6)A methyltransferase KIAA1429-mediated RNA metabolism during mouse follicular development. Cell Death Differ (2020) 27(8):2468–83. 10.1038/s41418-020-0516-1 PMC7370231 32094512

[10] Mathiyalagan P Adamiak M Mayourian J Sassi Y Liang Y Agarwal N. FTO-Dependent N(6)-Methyladenosine Regulates Cardiac Function During Remodeling and Repair. Circulation (2019) 139(4):518–32. 10.1161/circulationaha.118.033794 PMC6400591 29997116

[11] Zhang S Zhao BS Zhou A Lin K Zheng S Lu Z. m(6)A Demethylase ALKBH5 Maintains Tumorigenicity of Glioblastoma Stem-like Cells by Sustaining FOXM1 Expression and Cell Proliferation Program. Cancer Cell (2017) 31(4):591–606.e6. 10.1016/j.ccell.2017.02.013 28344040 PMC5427719

[12] Liu S Li G Li Q Zhang Q Zhuo L Chen X. The roles and mechanisms of YTH domain-containing proteins in cancer development and progression. Am J Cancer Res (2020) 10(4):1068–84. PMC7191095 32368386

[13] Lan Q Liu PY Haase J Bell JL Huttelmaier S Liu T. The Critical Role of RNA m(6)A Methylation in Cancer. Cancer Res (2019) 79(7):1285–92. 10.1158/0008-5472.Can-18-2965 30894375

[14] Jeck WR Sorrentino JA Wang K Slevin MK Burd CE Liu J. Circular RNAs are abundant, conserved, and associated with ALU repeats. RNA (New York NY) (2013) 19(2):141–57. 10.1261/rna.035667.112 PMC3543092 23249747

[15] Lei K Bai H Wei Z Xie C Wang J Li J. The mechanism and function of circular RNAs in human diseases. Exp Cell Res (2018) 368(2):147–58. 10.1016/j.yexcr.2018.05.002 29730164

[16] Kolde R Laur S Adler P Vilo J. Robust rank aggregation for gene list integration and meta-analysis. Bioinf (Oxford Engl) (2012) 28(4):573–80. 10.1093/bioinformatics/btr709 PMC3278763 22247279

[17] Tokar T Pastrello C Rossos AEM Abovsky M Hauschild AC Tsay M. mirDIP 4.1-integrative database of human microRNA target predictions. Nucleic Acids Res (2018) 46(D1):D360–d70. 10.1093/nar/gkx1144 PMC5753284 29194489

[18] Chen Y Yuan B Chen G Zhang L Zhuang Y Niu H. Circular RNA RSF1 promotes inflammatory and fibrotic phenotypes of irradiated hepatic stellate cell by modulating miR-146a-5p. J Cell Physiol (2020) 235(11):8270–82. 10.1002/jcp.29483 31960423

[19] Yang Z Wu L Wang A Tang W Zhao Y Zhao H. dbDEMC 2.0: updated database of differentially expressed miRNAs in human cancers. Nucleic Acids Res (2017) 45(D1):D812–d8. 10.1093/nar/gkw1079 PMC5210560 27899556

[20] Lian Q Wang S Zhang G Wang D Luo G Tang J. HCCDB: A Database of Hepatocellular Carcinoma Expression Atlas. Genomics Proteomics Bioinf (2018) 16(4):269–75. 10.1016/j.gpb.2018.07.003 PMC6205074 30266410

[21] Frye M Harada BT Behm M He C. RNA modifications modulate gene expression during development. Science (New York NY) (2018) 361(6409):1346–9. 10.1126/science.aau1646 PMC6436390 30262497

[22] Fu L Jiang Z Li T Hu Y Guo J. Circular RNAs in hepatocellular carcinoma: Functions and implications. Cancer Med (2018) 7(7):3101–9. 10.1002/cam4.1574 PMC6051148 29856133

[23] Tremblay MP Armero VE Allaire A Boudreault S Martenon-Brodeur C Durand M. Global profiling of alternative RNA splicing events provides insights into molecular differences between various types of hepatocellular carcinoma. BMC Genomics (2016) 17:683. 10.1186/s12864-016-3029-z 27565572 PMC5002109

[24] Chen SL Liu LL Wang CH Lu SX Yang X He YF. Loss of RDM1 enhances hepatocellular carcinoma progression via p53 and Ras/Raf/ERK pathways. Mol Oncol (2020) 14(2):373–86. 10.1002/1878-0261.12593 PMC6998392 31670863

[25] Zhao X Chen Y Mao Q Jiang X Jiang W Chen J. Overexpression of YTHDF1 is associated with poor prognosis in patients with hepatocellular carcinoma. Cancer Biomarkers section A Dis Markers (2018) 21(4):859–68. 10.3233/cbm-170791 PMC13078334 29439311

[26] Cui X Wang Z Li J Zhu J Ren Z Zhang D. Cross talk between RNA N6-methyladenosine methyltransferase-like 3 and miR-186 regulates hepatoblastoma progression through Wnt/beta-catenin signalling pathway. Cell Proliferation (2020) 53(3):e12768. 10.1111/cpr.12768 31967701 PMC7106953

[27] Zhou Y Yin Z Hou B Yu M Chen R Jin H. Expression profiles and prognostic significance of RNA N6-methyladenosine-related genes in patients with hepatocellular carcinoma: evidence from independent datasets. Cancer Manage Res (2019) 11:3921–31. 10.2147/cmar.S191565 PMC6503205 31118805

[28] Chen M Wei L Law CT Tsang FH Shen J Cheng CL. RNA N6-methyladenosine methyltransferase-like 3 promotes liver cancer progression through YTHDF2-dependent posttranscriptional silencing of SOCS2. Hepatol (Baltimore Md) (2018) 67(6):2254–70. 10.1002/hep.29683 29171881

[29] Lin X Chai G Wu Y Li J Chen F Liu J. RNA m(6)A methylation regulates the epithelial mesenchymal transition of cancer cells and translation of Snail. Nat Commun (2019) 10(1):2065. 10.1038/s41467-019-09865-9 31061416 PMC6502834

[30] Liu X Qin J Gao T Li C He B Pan B. YTHDF1 Facilitates the Progression of Hepatocellular Carcinoma by Promoting FZD5 mRNA Translation in an m6A-Dependent Manner. Mol Ther Nucleic Acids (2020) 22:750–65. 10.1016/j.omtn.2020.09.036 PMC7595883 33230473

[31] Bian S Ni W Zhu M Song Q Zhang J Ni R. Identification and Validation of the N6-Methyladenosine RNA Methylation Regulator YTHDF1 as a Novel Prognostic Marker and Potential Target for Hepatocellular Carcinoma. Front Mol Biosci (2020) 7:604766. 10.3389/fmolb.2020.604766 33363211 PMC7758441

[32] Hou J Zhang H Liu J Zhao Z Wang J Lu Z. YTHDF2 reduction fuels inflammation and vascular abnormalization in hepatocellular carcinoma. Mol Cancer (2019) 18(1):163. 10.1186/s12943-019-1082-3 31735169 PMC6859620

[33] Zhong L Liao D Zhang M Zeng C Li X Zhang R. YTHDF2 suppresses cell proliferation and growth via destabilizing the EGFR mRNA in hepatocellular carcinoma. Cancer Lett (2019) 442:252–61. 10.1016/j.canlet.2018.11.006 30423408

[34] Zhang L Huo Q Ge C Zhao F Zhou Q Chen X. ZNF143-mediated H3K9 trimethylation upregulates CDC6 by activating MDIG in hepatocellular carcinoma. Cancer Res (2020) 80(12):2599–611. 10.1158/0008-5472.Can-19-3226 32312832

[35] Chen CC Chen CY Ueng SH Hsueh C Yeh CT Ho JY. Corylin increases the sensitivity of hepatocellular carcinoma cells to chemotherapy through long noncoding RNA RAD51-AS1-mediated inhibition of DNA repair. Cell Death Dis (2018) 9(5):543. 10.1038/s41419-018-0575-0 29749376 PMC5945779

[36] Yang Z Li J Feng G Gao S Wang Y Zhang S. MicroRNA-145 Modulates N(6)-Methyladenosine Levels by Targeting the 3’-Untranslated mRNA Region of the N(6)-Methyladenosine Binding YTH Domain Family 2 Protein. J Biol Chem (2017) 292(9):3614–23. 10.1074/jbc.M116.749689 PMC5339747 28104805

[37] Hua S Lei L Deng L Weng X Liu C Qi X. miR-139-5p inhibits aerobic glycolysis, cell proliferation, migration, and invasion in hepatocellular carcinoma via a reciprocal regulatory interaction with ETS1. Oncogene (2018) 37(12):1624–36. 10.1038/s41388-017-0057-3 29335523

[38] Wang F Li L Piontek K Sakaguchi M Selaru FM. Exosome miR-335 as a novel therapeutic strategy in hepatocellular carcinoma. Hepatol (Baltimore Md) (2018) 67(3):940–54. 10.1002/hep.29586 PMC5826829 29023935

[39] Zhang L Geng Z Wan Y Meng F Meng X Wang L. Functional analysis of miR-767-5p during the progression of hepatocellular carcinoma and the clinical relevance of its dysregulation. Histochem Cell Biol (2020) 154(2):231–43. 10.1007/s00418-020-01878-6 32333091

[40] Wang X Gao J Zhou B Xie J Zhou G Chen Y. Identification of prognostic markers for hepatocellular carcinoma based on miRNA expression profiles. Life Sci (2019) 232:116596. 10.1016/j.lfs.2019.116596 31233760

[41] Wang YG Wang T Ding M Xiang SH Shi M Zhai B. hsa_circ_0091570 acts as a ceRNA to suppress hepatocellular cancer progression by sponging hsa-miR-1307. Cancer Lett (2019) 460:128–38. 10.1016/j.canlet.2019.06.007 31207319

