# Supplementary material for: Analysis and Validation of circRNA-miRNA Network in Regulating m6A RNA Methylation Modulators Reveals CircMAP2K4/miR-139-5p/YTHDF1 Axis Involving the Proliferation of Hepatocellular Carcinoma
Source: Front Oncol. 2021 Feb 23;11:560506. doi: 10.3389/fonc.2021.560506 (PMC7940687; doi:10.3389/fonc.2021.560506)
Supplement: Supplementary file 1 [file DataSheet_1.docx]

Supplementary Material

# Supplementary Figures and Tables

## Supplementary Figures

**FIGURE S1** The flowchart of study design.

**FIGURE S2** The analysis flowchart of HCC cases with complete expression data. N: normal, T: tumor.

**FIGURE S3** Relationship between m6A RNA methylation modulators and clinicopathological characteristics as well as GSEA in ICGC. (A) Comparison of YTHDF1 or HNRNPC expression level between different stages. (B) GSEA results showing the top 5 gene sets based on normalized enrichment score in YTHDF1 high or low expression groups.

**FIGURE S4** CircMAP2K4 has biding site for hsa-miR-139-5p. (A) Analysis of circMAP2K4, hsa-miR-139-5p and YTHDF1 expression in HCC cells. **P* < 0.05 vs. LO2 cells. (B) Agarose gel electrophoresis showed the amplified product of divergent primer (< >) or convergent primer (> <) for circMAP2K4 in cDNA or gDNA. (C) Validation of junction site of circMAP2K4 by Sanger sequencing (up panel). The circMAP2K4(has_circ_0007456) junction sequence (low panel) provided by Circular RNA Interactome (https://circinteractome.nia.nih.gov/). (D) qRT-PCR and Western blot analysis of YTHDF1 in HCC cells at 24h after transfection with YTHDF1 siRNAs. **P* < 0.05 vs. siNC. (E) qRT-PCR analysis of hsa-miR-139-5p in HCC cells at 24h after transfection with miRNA mimics. **P* < 0.05 vs. miR-NC. (F) qRT-PCR analysis of circMAP2K4 in HCC cells at 24h after transfection with circMAP2K4 expressing plasmid. **P* < 0.05 vs. circNC. (G) Luciferase activity in cells co-transfected with luciferase reporter containing the wild-type (WT) or mutated (MUT) hsa-miR-139-5p binding site of circMAP2K4 (left panel), wild-type (WT) or mutated (MUT) 3’UTR of YTHDF1 (right panel) and hsa-miR-139-5p mimics or control. **P* < 0.05 vs. miR-NC. siNC, siRNA negative control for YTHDF1, siYTHDF1: siRNA specifically against YTHDF1, miR-NC: miRNA negative control, miR-M: miRNA mimics, circNC: negative control for circMAP2K4 expressing plasmid.

## Supplementary Tables

**Table S1** siRNA sequences for YTHDF1

| siRNA | sense（5'to3'） | antisense（5'to3'） |
| --- | --- | --- |
| siNC | GGACAGUCAAAUCAGAGUGGC | UACUCUGAUUUGACUGUGGAG |
| siYTHDF1-1 | GGAGAAUAACGACAACAAACC | UUUGUUGUCGUUAUUCUCCAG |
| siYTHDF1-2 | GGCGUGUGUUCAUCAUCAAGA | UUGAUGAUGAACACACGCCCG |

**Table S2** Primers for qRT-PCR

| Gene | sense primer(5'to3') | antisense primer(5'to3') |
| --- | --- | --- |
| CircMAP2K divergent primer | GACTTCGGCATCAGTGGACA | ATCCCAGTGTTGTTCAGGGG |
| CircMAP2K convergent primer | TCCACAAACCAAGTGGGCAA | TGTCCACTGATGCCGAAGTC |
| YHTDF1 | ACCTGTCCAGCTATTACCCG | TGGTGAGGTATGGAATCGGAG |
| GAPDH | TGTGTCCGTCGTGGATCTGA | TTCGTGTTGAAGTCGCAGGAG |

**Table S3** The expression status of DEmiRNAs in different GEO datasets analyzed by dbDEMC 2.0.

| miRNA | GEO series | logFC | adj Pvalue | Status |
| --- | --- | --- | --- | --- |
| hsa-miR-136-5p | GSE6857 | 0.41280872 | 1.10E-19 | UP |
| hsa-miR-136-5p | GSE39678 | 0.763030302 | 0.034426569 | UP |
| hsa-miR-136-5p | GSE36915 | -1.172513831 | 0.01683755 | DOWN |
| hsa-miR-139-5p | GSE6857 | -0.302154622 | 1.12E-10 | DOWN |
| hsa-miR-139-5p | GSE22058 | -0.244880577 | 9.58E-18 | DOWN |
| hsa-miR-139-5p | GSE21362 | -1.41751349 | 2.18E-08 | DOWN |
| hsa-miR-139-5p | GSE39678 | -0.710582086 | 0.033598039 | DOWN |
| hsa-miR-139-5p | GSE40744 | -1.27814452 | 0.016800728 | DOWN |
| hsa-miR-139-5p | GSE36915 | -1.331940069 | 9.11E-08 | DOWN |
| hsa-miR-195-5p | GSE10694 | -0.613693182 | 6.03E-06 | DOWN |
| hsa-miR-195-5p | GSE10694 | -0.551427705 | 0.001300075 | DOWN |
| hsa-miR-195-5p | GSE6857 | -0.317332536 | 1.63E-08 | DOWN |
| hsa-miR-195-5p | GSE22058 | -0.351304774 | 7.09E-18 | DOWN |
| hsa-miR-195-5p | GSE21362 | -0.875910851 | 1.64E-06 | DOWN |
| hsa-miR-195-5p | GSE36915 | -0.547738805 | 0.002653181 | DOWN |
| hsa-miR-335-5p | GSE6857 | -0.316002913 | 1.41E-19 | DOWN |
| hsa-miR-335-5p | GSE36915 | -0.478068258 | 0.009595524 | DOWN |
| hsa-miR-369-3p | GSE6857 | 1.044093664 | 8.17E-90 | UP |
| hsa-miR-369-3p | GSE22058 | 0.084922976 | 0.015099908 | UP |
| hsa-miR-376c-3p | GSE6857 | 0.114657429 | 0.01596458 | UP |
| hsa-miR-381-3p | GSE22058 | 0.08418033 | 0.002684762 | UP |
| hsa-miR-424-5p | GSE10694 | -0.556810711 | 0.000515503 | DOWN |
| hsa-miR-424-5p | GSE22058 | -0.327425589 | 1.78E-28 | DOWN |
| hsa-miR-424-5p | GSE21362 | -1.521234114 | 2.53E-10 | DOWN |
| hsa-miR-424-5p | GSE39678 | -1.014187604 | 0.006119441 | DOWN |
| hsa-miR-424-5p | GSE36915 | -0.417786307 | 0.000885717 | DOWN |
| hsa-miR-497-5p | GSE10694 | -0.277088475 | 0.003308009 | DOWN |
| hsa-miR-497-5p | GSE10694 | -0.260898749 | 0.02456939 | DOWN |
| hsa-miR-497-5p | GSE21362 | -0.806115107 | 0.000168397 | DOWN |
| hsa-miR-497-5p | GSE36915 | -0.932875652 | 0.006286864 | DOWN |
| hsa-miR-511-5p | GSE39678 | 1.176830781 | 0.001019308 | UP |
| hsa-miR-511-5p | GSE36915 | -1.085903588 | 6.04E-05 | DOWN |

**Table S4** The expression level of DEmRNAs in different GEO datasets analyzed by HCCDB.

| Data | Type | HNRNPC | | YTHDF1 | | YTHDF2 | |
| --- | --- | --- | --- | --- | --- | --- | --- |
|  |  | *P* | Mean | *P* | Mean | *P* | Mean |
| GSE22058 | HCC | 0.2581 | 9.844 | 0.01141 | 10.42 | 0.8524 | 9.816 |
|  | Adjacent |  | 9.801 |  | 10.32 |  | 9.826 |
| GSE25097 | HCC | 0.9286 | 1.641 | 6.90E-21 | 7.532 | 0.3089 | 1.706 |
|  | Adjacent |  | 1.644 |  | 6.694 |  | 1.753 |
|  | Cirrhotic |  | 1.729 |  | 7.261 |  | 1.659 |
|  | Healthy |  | 1.48 |  | 7.945 |  | 2.05 |
| GSE36376 | HCC | 5.70E-56 | 7.856 | 3.92E-100 | 8.948 | 7.91E-17 | 8.492 |
|  | Adjacent |  | 7.385 |  | 8.089 |  | 8.17 |
| GSE14520 | HCC | 1.83E-61 | 9.057 |  |  | 9.16E-13 | 7.524 |
|  | Adjacent |  | 8.443 |  |  |  | 7.155 |
| GSE10143 | HCC | 0.001084 | 12.13 |  |  |  |  |
|  | Adjacent |  | 11.59 |  |  |  |  |
| GSE46444 | HCC | 0.2888 | 7.113 | 0.6392 | 6.87 | 0.6548 | 6.598 |
|  | Adjacent |  | 7.283 |  | 6.781 |  | 6.683 |
| GSE54236 | HCC | 0.0006015 | 12.13 | 2.41E-07 | 10.35 | 0.2624 | 11.27 |
|  | Adjacent |  | 11.97 |  | 10.12 |  | 11.22 |
| GSE63898 | HCC | 0.1757 | 8.859 | 3.01E-10 | 5.836 | 0.0000548 | 8.631 |
|  | Adjacent |  | 8.818 |  | 5.657 |  | 8.466 |
| GSE64041 | HCC | 0.02525 | 10.03 | 0.0000878 | 9.38 | 0.0000446 | 8.477 |
|  | Adjacent |  | 9.871 |  | 9.219 |  | 8.352 |
| GSE76427 | HCC | 5.03E-07 | 9.18 | 5.55E-11 | 10.04 | 0.06209 | 9.677 |
|  | Adjacent |  | 8.982 |  | 9.693 |  | 9.558 |
