# Supplementary figures and images for: Analysis and Validation of circRNA-miRNA Network in Regulating m6A RNA Methylation Modulators Reveals CircMAP2K4/miR-139-5p/YTHDF1 Axis Involving the Proliferation of Hepatocellular Carcinoma
Source: Front Oncol. 2021 Feb 23;11:560506. doi: 10.3389/fonc.2021.560506 (PMC7940687; doi:10.3389/fonc.2021.560506)

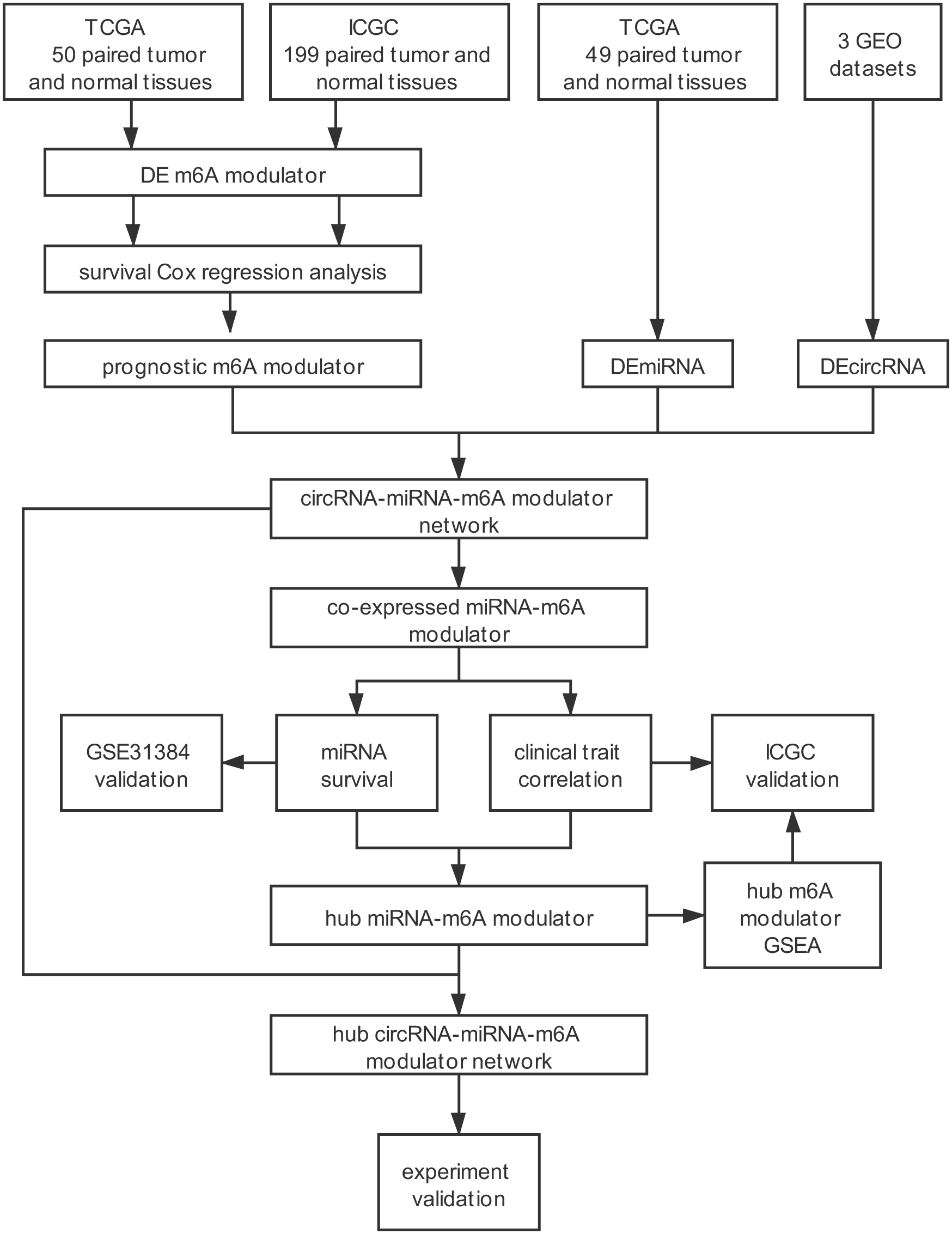

Supplement: Supplementary file 2 [file Image_1.jpeg]

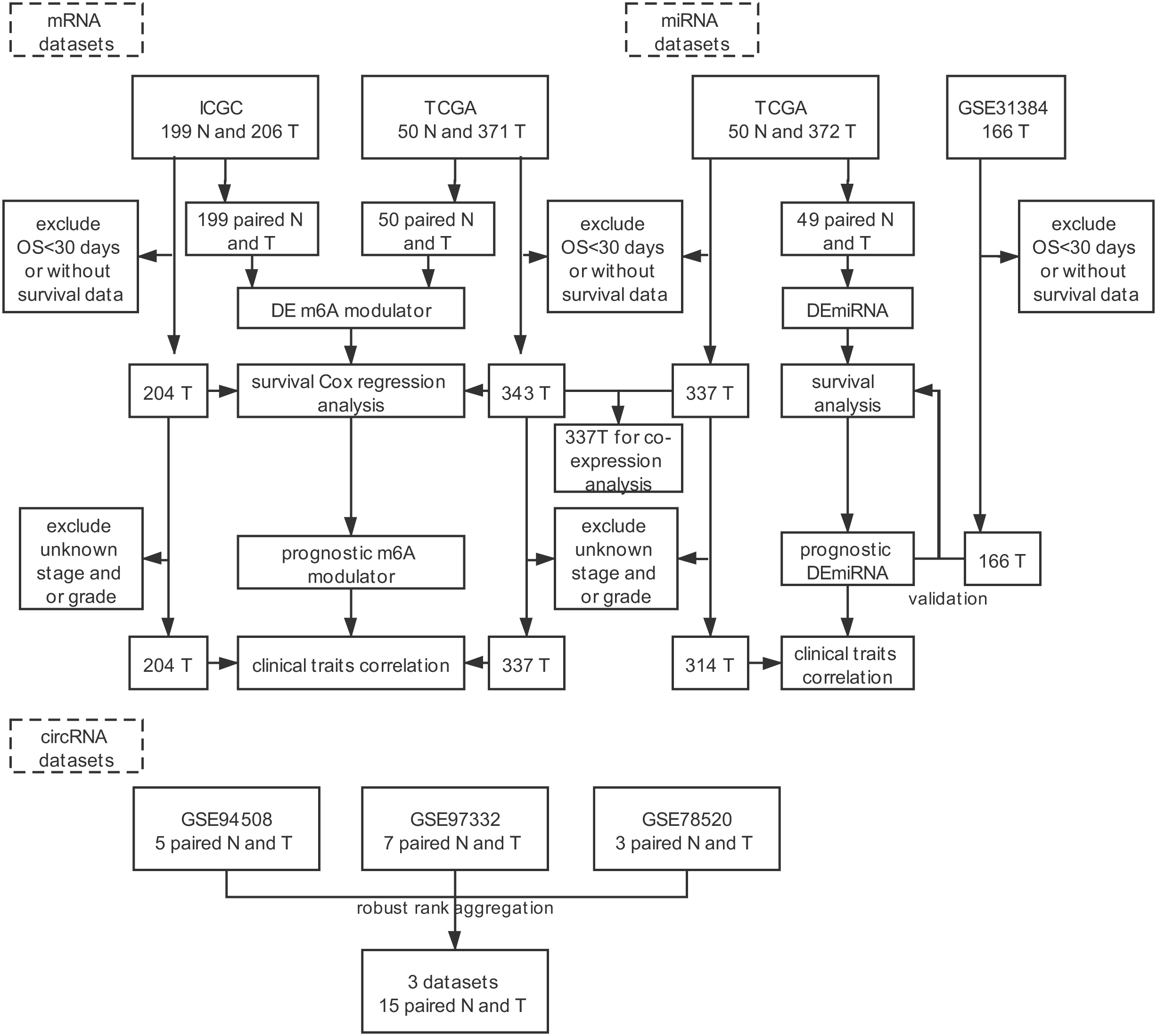

Supplement: Supplementary file 3 [file Image_2.jpeg]

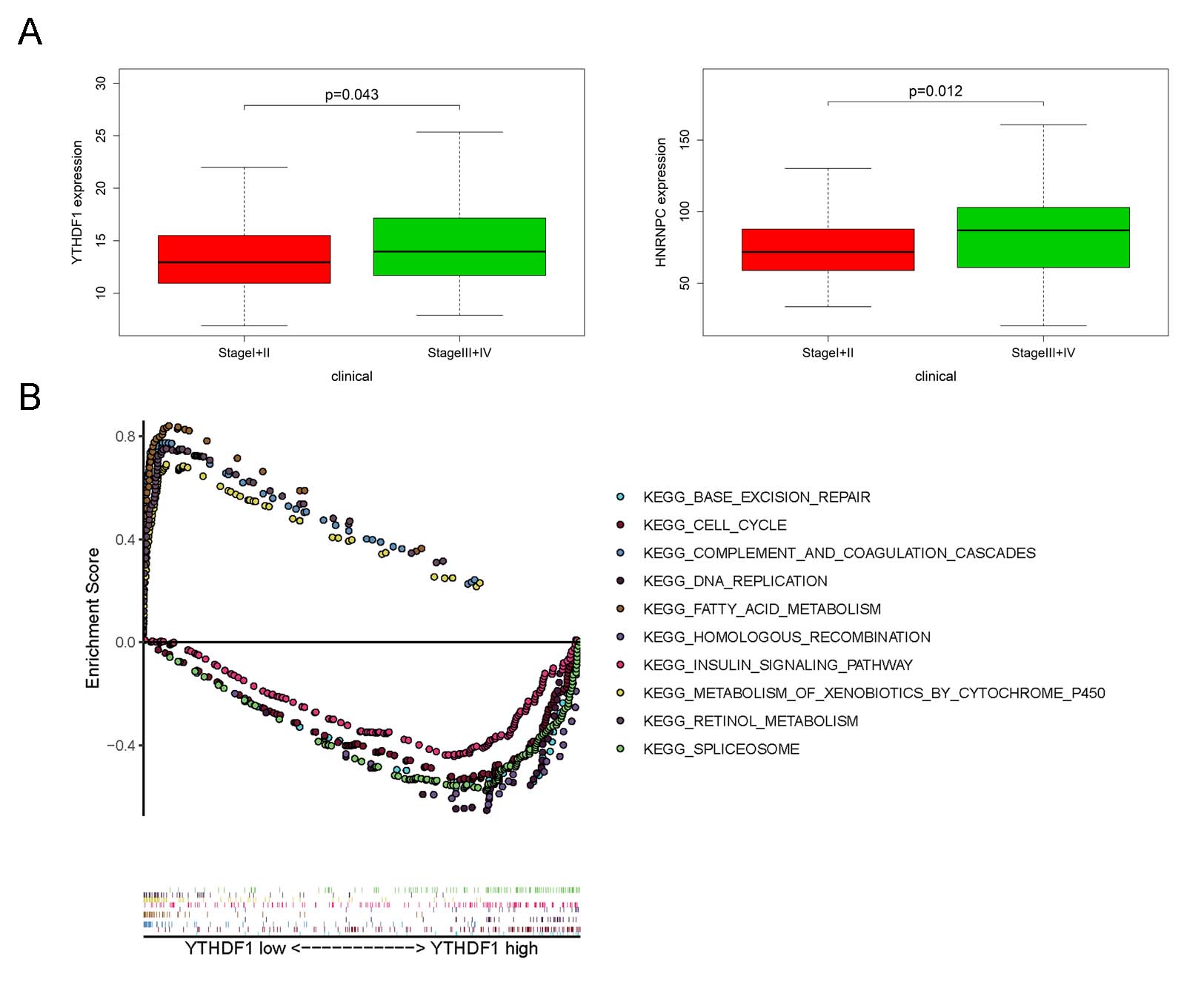

Supplement: Supplementary file 4 [file Image_3.jpeg]

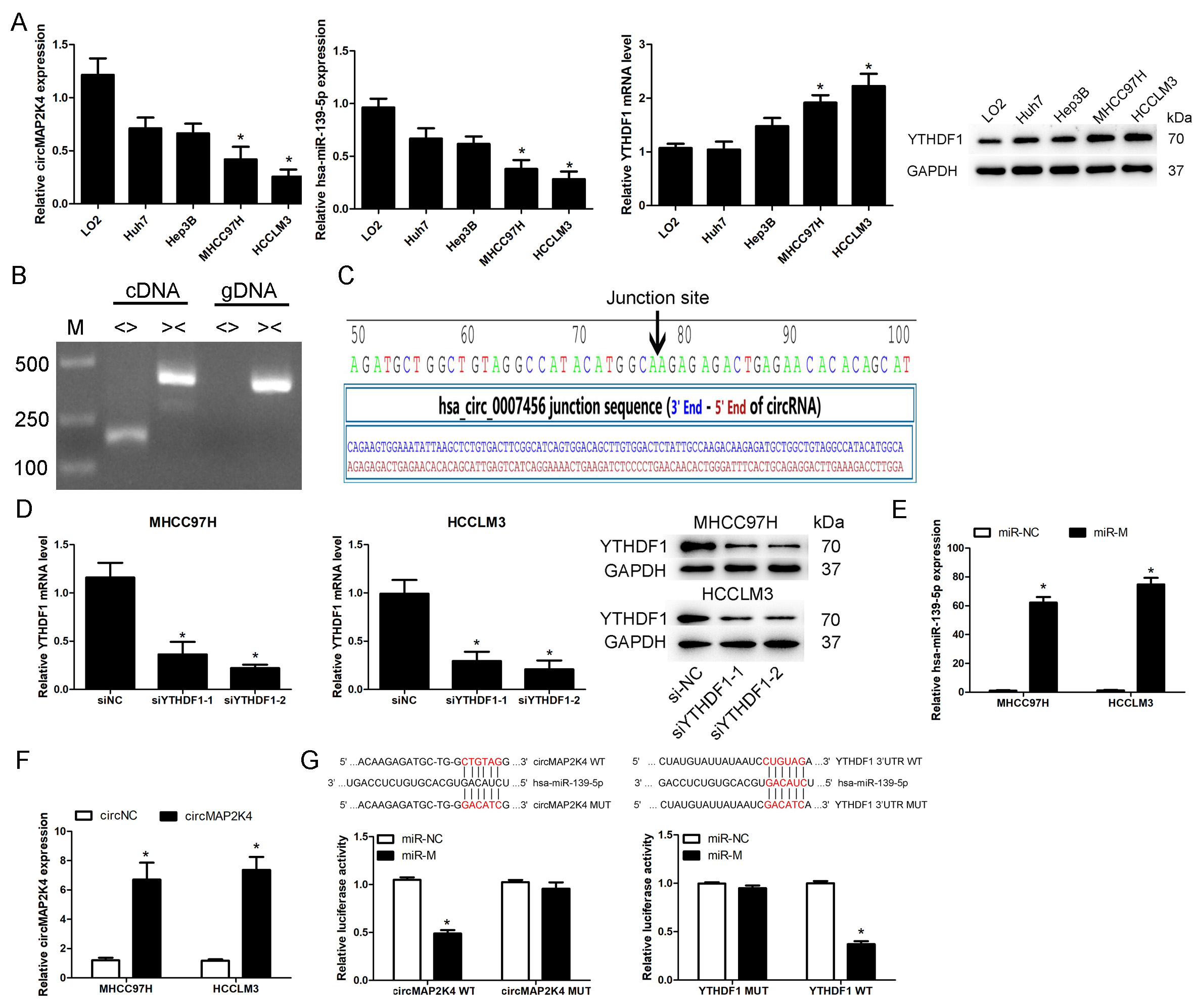

Supplement: Supplementary file 5 [file Image_4.jpeg]
